# The coproporphyrin ferrochelatase of *Staphylococcus aureus*: mechanistic insights into a regulatory iron-binding site

**DOI:** 10.1042/BCJ20170362

**Published:** 2017-10-10

**Authors:** Charlie Hobbs, James D. Reid, Mark Shepherd

**Affiliations:** 1 School of Biosciences, University of Kent, Canterbury CT2 7NJ, U.K.; 2 Department of Chemistry, University of Sheffield, Sheffield S3 7HF, U.K.

**Keywords:** coproporphyrinogen, ferrochelatase, haem biosynthesis

## Abstract

The majority of characterised ferrochelatase enzymes catalyse the final step of classical haem synthesis, inserting ferrous iron into protoporphyrin IX. However, for the recently discovered coproporphyrin-dependent pathway, ferrochelatase catalyses the penultimate reaction where ferrous iron is inserted into coproporphyrin III. Ferrochelatase enzymes from the bacterial phyla Firmicutes and Actinobacteria have previously been shown to insert iron into coproporphyrin, and those from *Bacillus subtilis* and *Staphylococcus aureus* are known to be inhibited by elevated iron concentrations. The work herein reports a *K*_m_ (coproporphyrin III) for *S. aureus* ferrochelatase of 1.5 µM and it is shown that elevating the iron concentration increases the *K*_m_ for coproporphyrin III, providing a potential explanation for the observed iron-mediated substrate inhibition. Together, structural modelling, site-directed mutagenesis, and kinetic analyses confirm residue Glu271 as being essential for the binding of iron to the inhibitory regulatory site on *S. aureus* ferrochelatase, providing a molecular explanation for the observed substrate inhibition patterns. This work therefore has implications for how haem biosynthesis in *S. aureus* is regulated by iron availability.

## Introduction

**Figure 1.**
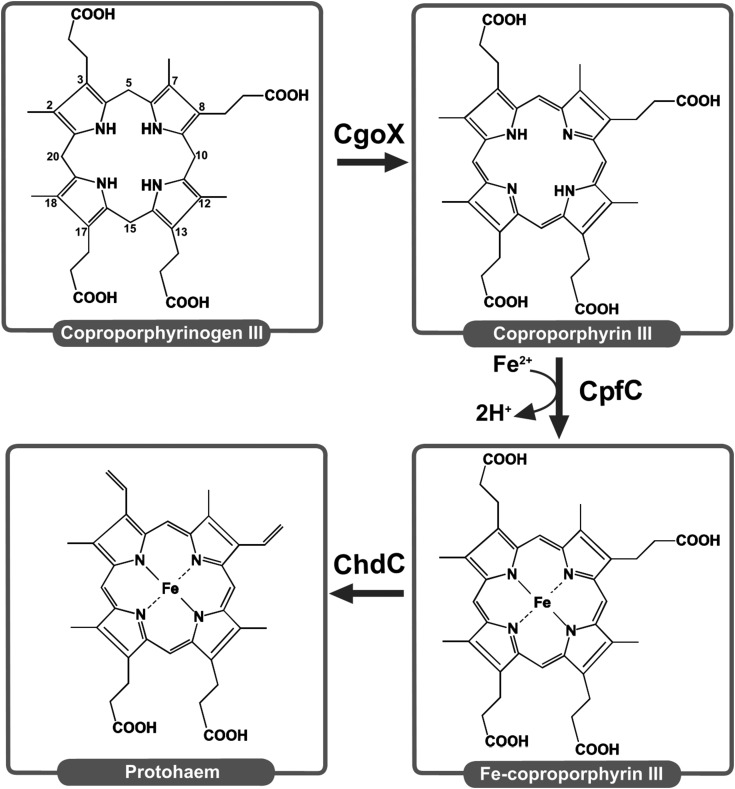
The coproporphyrin-dependent pathway of haem synthesis. This figure depicts a recently discovered route for haem synthesis in Firmicutes and Actinobacteria catalysed by coproporphyrinogen oxidase (CgoX), CpfC, and coprohaem decarboxylase (ChdC). These new abbreviations are introduced in a recent review [3].

Ferrochelatase enzymes catalyse the insertion of metal ions into porphyrin rings. The insertion of ferrous iron (Fe^2+^) into protoporphyrin IX is the final step in the well-characterised classical haem synthesis pathway. However, bacterial species in the Firmicutes and Actinobacteria Phyla (*Bacillus subtilis*, *Mycobacterium tuberculosis*, *Propionibacterium acnes*, *S. aureus*) have been shown to utilise a ‘coproporphyrin-dependent’ pathway [1,2], where Fe^2+^ insertion into coproporphyrin III is the penultimate step (Figure 1). Branching and regulation of these bacterial haem biosynthetic pathways are discussed in a recent review that suggests a series of new abbreviations based on enzyme activities [3]. These new abbreviations provide a useful way of discriminating between protoporphyrin ferrochelatase (PpfC) and coproporphyrin ferrochelatase (CpfC), and this nomenclature is adopted herein in place of the traditional HemH abbreviation.

Ferrochelatases are membrane-bound proteins in eukaryotes and many prokaryotes, but Gram-positive bacteria have been shown to encode a soluble form of the protein [4–6]. All of these forms of ferrochelatase have been shown to insert a variety of divalent metals into the centre of porphyrin molecules, with zinc being the most common metal used in *in vitro* assays due to being more stable in an aerobic environment than the natural Fe^2+^ substrate [7] and allows for fluorescence-based assays to be utilised. Catalytic rates for ferrochelatases vary with *k*_cat_ values typically ranging from 0.11 to 15.3 min^−1^, although *S. aureus* CpfC was recently reported to have a *k*_cat_ of 165 min^−1^ using coproporphyrin III and Fe^2+^ as substrates [1]. Parallel experiments performed for the *B. subtilis* CpfC gave a *k*_cat_ of 78 min^−1^, which compares to another report of 0.11 min^−1^ [2]. This recent work also identified 0.8 µM as the threshold concentration of Fe^2+^ above which inhibition occurs for the *B. subtilis* and *S. aureus* enzymes, which represents an interesting feature unique to coproporphyrin-dependent ferrochelatases.

While bacterial and mammalian ferrochelatase enzymes generally exhibit poor sequence identity (e.g. <10% between human and *B. subtilis*), the secondary and tertiary structures are similar in the core region [8]. However, human and yeast PpfC enzymes have an extended C-terminal region compared with *B. subtilis* CpfC. The human enzyme ligates a [2Fe–2S] iron–sulphur cluster in this region, which is not present in the *S. cerevisiae* enzyme. This cluster is ligated by one residue in the core of the protein (Cys 196) and three in the extended C-terminal region (Cys 403, 406, 411), and at the time was thought to be responsible for anchoring the extended C-terminal to the rest of the monomer, and was also suggested to be indirectly involved in stabilising the dimer form of the protein [8]. *S. cerevisiae* does not contain the necessary residues in the same location to form a cluster and the dimer stability is reportedly not affected [9]. Some ferrochelatases from Gram-positive bacteria have been shown to ligate [2Fe–2S] clusters via an extra insertion, although loss of this cluster did not result in a total loss of activity [10] in contrast with the [2Fe–2S] cluster of the human enzyme [11]. The *in vivo* role for these cofactors remains poorly understood.

Ferrochelatase structures exhibit a degree of variability with respect to the residues present in the substrate binding cleft. For eukaryotic PpfC enzymes a series of leucine residues form part of the hydrophobic lip that covers the active site pocket, which is not present in the *B. subtilis* enzyme. The Tyr13 residue in *B. subtilis* CpfC, which has been determined to aid the conserved His183 residue in stabilisation of the B ring of the porphyrin macrocycle, is not present in the membrane-associated ferrochelatases [12]. The residue in this position in the other enzymes is a methionine whose role is not entirely understood [8,9]. The tyrosine residue in *B. subtilis* has been implicated in a metal-binding function in the catalytic metal-binding site along with the conserved histidine and glutamic acid residues [12,13] and it is possible that the methionine residue in this position aids in the insertion of metals into the porphyrin ring in a similar manner to the tyrosine residue found in the soluble form of ferrochelatase. The amino acid at this position has also been linked to the specificity of metal chelation, with an Y13M mutation of the *B. subtilis* enzyme resulting in a more rapid insertion of Co^2+^ and a complete loss of Cu^2+^ utilisation [14].

Soluble ferrochelatases have been crystallised with a variety of metals present in the metal-binding cleft, and have been shown to have two separate sites for metal binding: one close to the porphyrin-binding site stabilised by the conserved His183 and one closer to the surface of the protein ∼7 Å from the H183 residue [12]. In *B. subtilis* CpfC, this outer site has been shown to bind Mg^2+^ ions, which are co-ordinated by the residues Arg46, Glu268, and Glu272 [12]. This particular metal site has been reported to provide a Mg^2+^-mediated stimulation of zinc insertion into deuteroporphyrin IX, with mutation of the Glu272 residue resulting in abolition of the stimulatory effects of Mg^2+^ [13]. The mechanism for this stimulation was proposed to be due to repulsion between metal ions present at both sites pushing the inserted metal at the inner (catalytic)-binding site closer to the porphyrin ring [13]. Such a regulatory metal site has not been investigated in the human enzyme, and it seems unlikely that *S. cerevisiae* PpfC could bind metals at this site due to the presence of a glycine residue instead of a glutamate residue at position 322 [9]. However, it has been suggested that *S. cerevisiae* PpfC can bind the inhibitor Cd^2+^ at two sites, with one site slightly further away from the active site than the outer metal-binding site for *B. subtilis* CpfC. Human PpfC is susceptible to substrate inhibition when zinc is the metal ion substrate. This zinc inhibition is more pronounced at higher concentrations of the porphyrin substrate, suggesting that an inhibitory metal ion binds to the enzyme–product complex [15]. Residues that are predicted to be predominantly involved in metal binding in *B. subtilis*, where Zn^2+^ and Cd^2+^ have been observed, are the conserved residues H183 and E264 and the unique Y13 residue that resides on the other side of the porphyrin-binding site [13]. Cd^2+^ has been shown to also bind to the nearby H262, which is located underneath the porphyrin substrate and is proposed to inhibit CpfC by hampering the binding of porphyrin substrate [13].

The current work investigates the kinetic properties of CpfC from the human pathogen *S. aureus* using the native substrates Fe^2+^ and coproporphyrin III. Since iron has dramatic inhibitory properties for this class of CpfCs [1], this study aims to test the hypothesis that iron inhibition is mediated by the regulatory metal-binding site previously shown to bind Mg^2+^ in the *B. subtilis* enzyme [12,13]. This work has implications for how staphylococci respond to nutritional immunity (e.g. metal sequestration) encountered during infection.

## Experimental procedures

### Cloning, overexpression, and purification of *S. aureus* ferrochelatase

The *cpfC* (*hemH*) gene was amplified from *S. aureus* strain USA300 [16] via colony PCR using Q5 polymerase (NEB) and cloned into the *Nhe*I/*Hin*dIII sites of plasmid pTrcHis (ThermoFischer), and the correct sequence of the resultant pTrcHis-*Sa-cpfC* vector was confirmed by Sanger sequencing. *Escherichia coli* JM109 cells (Sigma–Aldrich) were transformed with pTrcHis-*Sa-cpfC* and a 10 ml LB overnight culture of this expression strain was used to inoculate 1 l of Circlegrow medium in a 2-l baffled flask (125 µg ml^−1^ ampicillin was included throughout for plasmid selection). Cultures were grown for 24 h at 37°C and 160 rpm and cells were harvested (4 k rpm, 4°C, 20 min) and stored at −20°C. Cell pellets from 1 l cultures were re-suspended in protein storage buffer (30 ml) containing 50 mM Tris/MOPS pH 8.0, 100 mM KCl, 1% sodium cholate. Cell suspensions were sonicated (6 × 30 s on ice at 10 µ, with 30 s intervals between bursts), and cell debris was removed via centrifugation (18 k rpm, 4°C, 20 min). The supernatant was applied to a column containing Talon metal affinity resin (2.5 ml bed volume) equilibrated with 5 column volumes of protein storage buffer. The column was washed with 20 column volumes of protein storage buffer followed by 5 column volumes of wash buffer (protein storage buffer including 15 mM imidazole), and purified protein was eluted with 5 column volumes of elution buffer (protein storage buffer including 300 mM imidazole). Purified protein was concentrated to <1 ml using a 10 kDa spin concentrator (Millipore) and imidazole was removed using a PD10 desalting column (GE Healthcare) equilibrated with protein storage buffer.

### Assay of *S. aureus* ferrochelatase activity

Purified CpfC was assayed using coproporphyrin III (Frontier Scientific) as the porphyrin substrate and ferrous iron as the inserted metal ion. Coproprophyrin III powder was solubilised using a few drops of ammonium hydroxide and was then diluted in assay buffer (0.1 M Tris–HCl pH 8.1, 0.5% sodium cholate, 0.5% Tween 20, 1 mM β-mercaptoethanol). Coproporphyrin III stock solutions were quantified in 0.1 M HCl spectrophotometrically using an extinction coefficient of *ε*_399.5 _= 489 mM^−1 ^cm^−1^ [17]. Ferrous iron solutions were quantified spectrophotometrically using ferrozine solution and an extinction coefficient of *ε*_562_ = 27.9 mM^−1 ^cm^−1^ [18]. Iron chelation was measured using a continuous assay essentially, as previously described [10]. Activity of 50 nM CpfC was monitored using a Cary 60 spectrophotometer at 30°C. The reactions were initiated by the addition of enzyme, which was preincubated at 30°C, and the depletion of coproporphyrin III was monitored at 392 nm for 3 min (*ε*_392_ = 115 mM^−1 ^cm^−1^, [1]). All assays contained 0.1 M Tris–HCl pH 8.1, 0.5% sodium cholate, 0.5% Tween 20, 1 mM β-mercaptoethanol. The steady-state rates were estimated using linear regression of the timecourse at the start of the reaction. Michaelis plots (*v* vs. [S]) were fitted to the Michaelis–Menten equation using non-linear regression. *K*_i,app_ values were calculated by fitting *v* vs. [inhibitor] data to a single-binding site model described by eqn (1) [19], where no mechanistic assumptions are made to perform a quantitative assessment of the key features of inhibition

1 $$v=v_{\min}+\frac{v_{\mathrm{control}}-v_{\min}}{{1+([I]/K_{i},\mathrm{app})}^{n}}$$

where *v* is the enzymatic rate, *v*_control_ is the rate in the absence of inhibitor, *v*_min_ is the rate in the presence of saturating inhibitor, [I] is the inhibitor concentration, *K*_i,app_ is the apparent inhibition constant defining the inhibitor concentration that elicits 50% of the total inhibition, and *n* is the Hill coefficient.

### Structural modelling of *S. aureus* ferrochelatase

A structural model for *S. aureus* CpfC was generated using the RaptorX server [20,21] with *B. subtilis* CpfC as a template (PDBid = 1AK1, α-carbon RMSD = 0.23 Å). The *S. aureus* CpfC model was superposed (α-carbon RMSD = 0.66 Å) onto a Mg^2+^-bound and porphyrin-bound structure of *B. subtilis* CpfC (PDBid = 1C1H), and the native substrate coproporphyrin III was modelled into the active site in place of *N*-methyl mesoporphyrin via superposition using the CCP4MG software.

## Results

### Measurement of kinetic constants for *S. aureus* ferrochelatase

**Figure 2.**
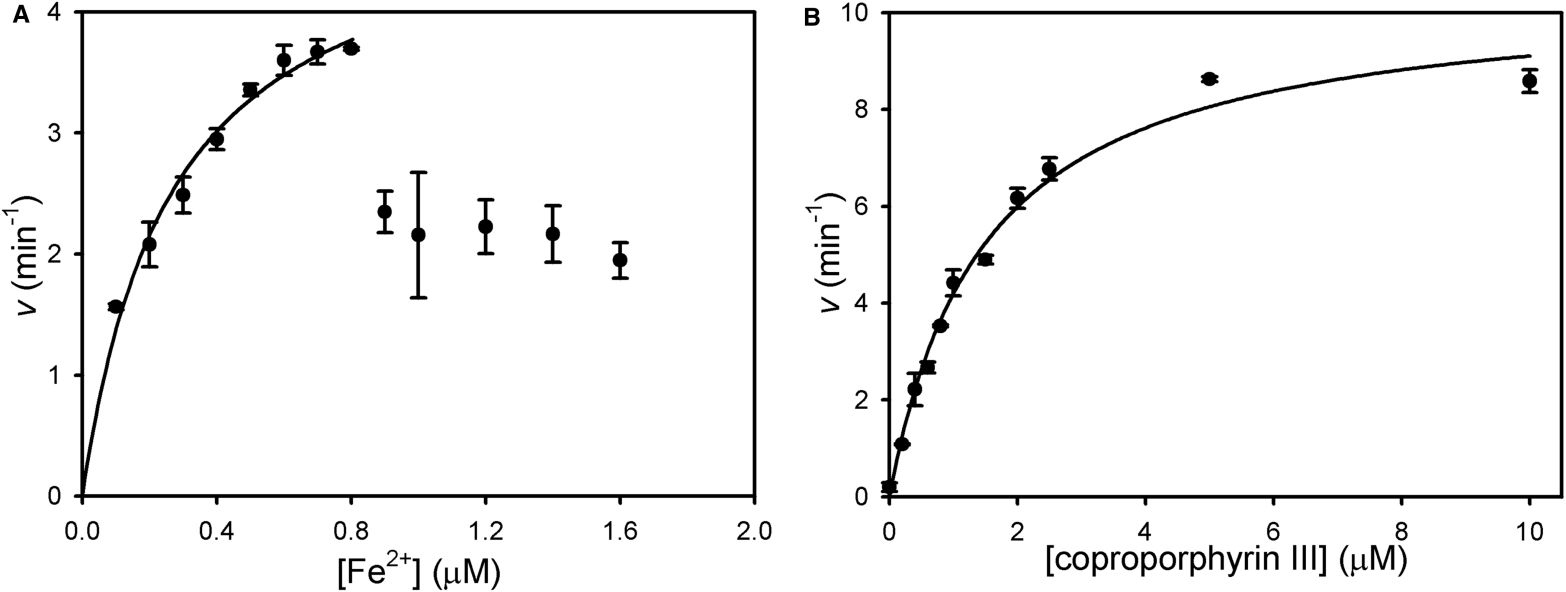
Kinetic analysis of *S. aureus* ferrochelatase. (**A**) 50 nM CpfC was assayed in the presence of varying concentrations of iron and a fixed concentration of 1 µM of coproporphyrin. Data collected at 0–0.8 µM iron were fitted to a single rectangular hyperbola to provide an estimate of the apparent *K*_m_ for iron (*K*_app_ = 0.27 ± 0.04). (**B**) 50 nM CpfC was assayed in the presence of varying concentrations of coproprophyrin III and a fixed iron concentration of 0.7 µM (below the 0.8 µM inhibitory iron concentration). Data were fitted to a single rectangular hyperbola via non-linear regression: *k*_cat_ = 10.5 ± 0.4 min^−1^, *K*_app_ = 1.5 ± 0.2 µM.

Since much of the previous work on CpfCs has been performed with non-native substrates (i.e. Zn^2+^, protoporphyrin IX, and deuteroporphyrin IX), it was of interest to define the kinetic constants for the *in vivo* substrates Fe^2+^ and coproporphyrin III. Kinetic assays where [Fe^2+^] was varied (Figure 2A) demonstrate substrate inhibition above 0.8 µM, and the data below this threshold value were fitted to the Michaelis equation to provide an estimate of 0.27 ± 0.04 µM for an apparent *K*_m_ for iron binding at the inner catalytic metal site. Varying [coproprophyrin III] at a fixed concentration of 0.7 µM Fe^2+^ (Figure 2B) provides the first estimate of an apparent *K*_m_ for coproprophyrin III as 1.5 ± 0.2 µM, and *k*_cat_ was fitted as 10.5 ± 0.4 min^−1^ at this sub-inhibitory Fe^2+^ concentration.

### Elevated iron increases the *K*_m_ for coproporphyin III

**Figure 3.**
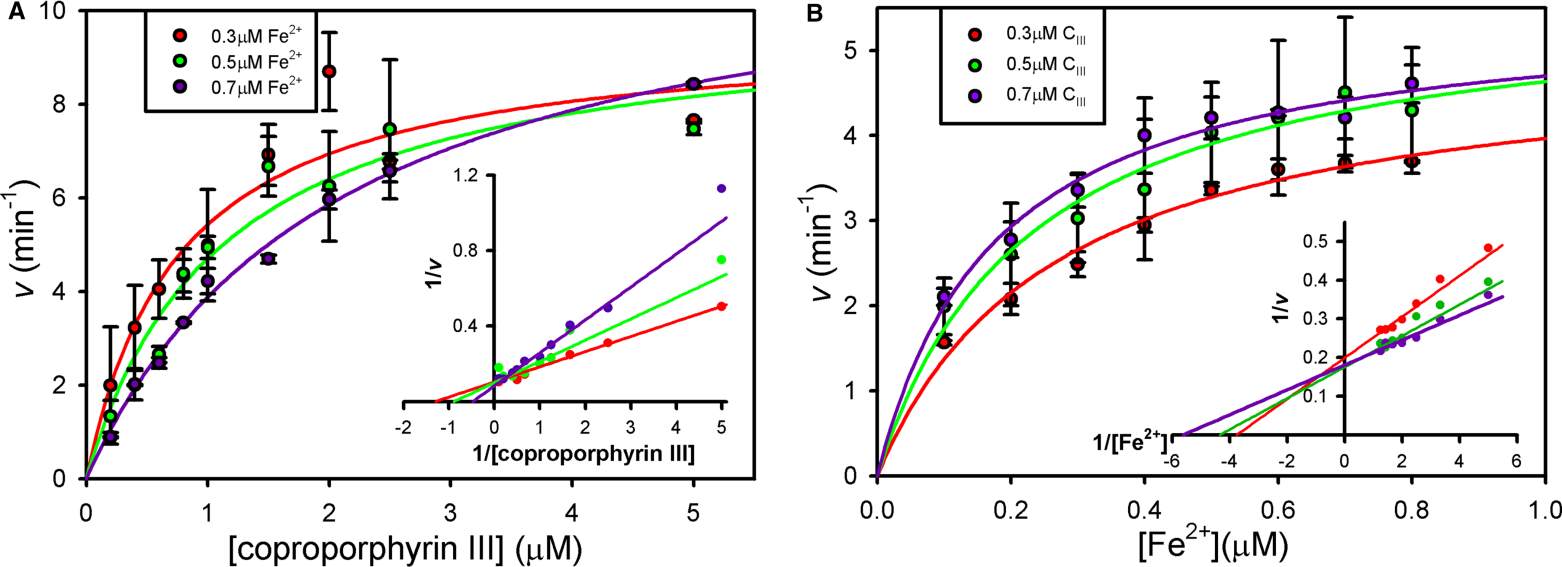
Opposing kinetic patterns for the two ferrochelatase substrates. Primary Michaelis–Menten plots were generated with fixed concentrations of iron (**A**) and coprorporphyrin III (**B**), and data were fitted to single rectangular hyperbolae via non-linear regression. Lineweaver–Burk plots (insets) are shown to highlight differences in kinetic patterns between panels (**A**) and (**B**). To avoid well-known problems with these secondary plots, trend lines were generated using the kinetic constants obtained from non-linear regression of the untransformed data.

To gain an insight into the influence of iron upon coproporphyrin III binding, kinetic plots were produced for each substrate at fixed concentrations of the other substrate (Figure 3). Firstly, varying the [coproporphyrin III] in the presence of fixed (sub-inhibitory) concentrations of Fe^2+^ revealed that increasing [Fe^2+^] increases the apparent *K*_m_ for coproporphyrin (Figure 3A). Performing the converse experiment with variation of [Fe^2+^] in the presence of fixed concentrations of coproporphyrin III revealed a more conventional pattern of increasing the concentration of coproporphyrin III also decreases the apparent *K*_m_ for Fe^2+^ (Figure 3B).

### Structural modelling of *S. aureus* ferrochelatase reveals a regulatory metal-binding site

**Figure 4.**
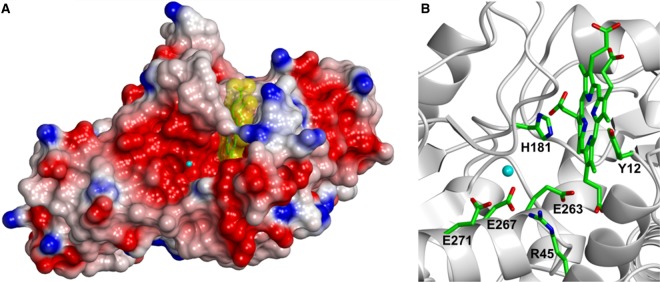
Structural modelling of bound coproprophyrin and a regulatory metal-binding site for *S. aureus* ferrochelatase. (**A**) Surface electrostatics view of a *S. aureus* CpfC structural model generated using the RaptorX server with *B. subtilis* CpfC as a template (PDBid = 1AK1, α-carbon RMSD = 0.3 Å). The *S. aureus* CpfC model was superposed (α-carbon RMSD = 0.66 Å) onto an Mg^2+^-bound (cyan) and porphyrin-bound structure of *B. subtilis* CpfC (PDBid = 1C1H), and the native substrate coproporphyrin III (yellow) was modelled into the active site in place of *N*-methyl mesoporphyrin. (**B**) Cartoon representation of the active site of *S. aureus* CpfC showing conserved residues implicated in metal binding at an outer regulatory site (E271, E267, R45) and an inner catalytic site (E263, Y12, H181).

Based on the data in Figure 3, it was hypothesised that *S. aureus* CpfC has a regulatory metal-binding site, and the most simple explanation would be that this metal site is at the same location as the Mg^2+^-binding site in *B. subtilis* CpfC. The only difference is that this site would be inhibitory when occupied by iron, as opposed to stimulatory when occupied by Mg^2+^ [13]. To investigate the local environment of this putative regulatory metal site, a structural model for *S. aureus* CpfC was generated using the *B. subtilis* CpfC crystal structure as a template (Figure 4). The model and the template were superposed and the Mg^2+^ ion from the *B. subtilis* structure was shown in the same spatial location in the *S. aureus* model (Figure 4A). The metal-binding residues were fully conserved between species, with residues Glu271, Glu267, and Arg45 in the *S. aureus* model all mapping adjacent to the outer regulatory metal site (Figure 4B).

### Magnesium potentiates Fe^2+^-mediated inhibition of *S. aureus* ferrochelatase

**Figure 5.**
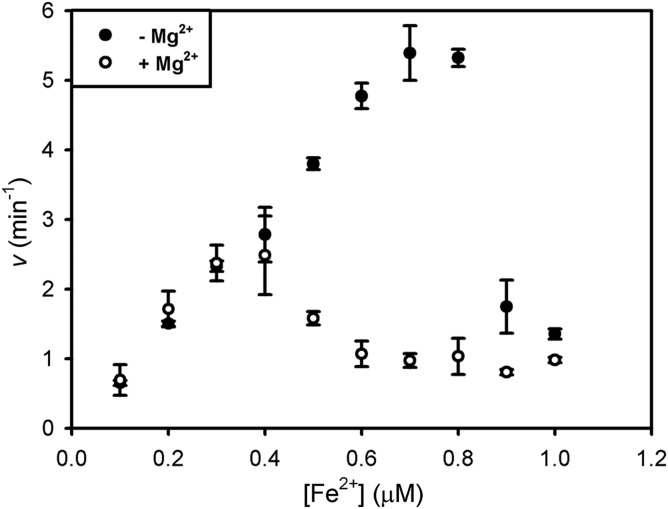
Impact of magnesium upon ferrochelatase kinetics. *v* vs. [Fe^2+^] plots were generated for *S. aureus* CpfC assayed in the presence (○) and absence (●) of 4 mM Mg^2+^. Fixed concentrations of 50 nM enzyme and 2 µM coproporphyrin III were used, with the Fe^2+^ concentration being varied.

Since Mg^2+^ has previously been used to investigate the regulatory metal site in *B. subtilis* CpfC and was shown to stimulate chelatase activity [13], it was of interest to determine whether Mg^2+^ could also be used as a molecular probe to study *S. aureus* CpfC. To investigate the effect of Mg^2+^ upon the Fe^2+^ kinetics, iron was varied in the presence and absence of Mg^2+^ (Figure 5). Surprisingly, the addition of Mg^2+^ potentiates chelatase inhibition, lowering the threshold concentration of Fe^2+^ required for substrate inhibition.

### Loss of the regulatory-binding site abolishes inhibition by Mg^2+^ or Fe^2+^

**Figure 6.**
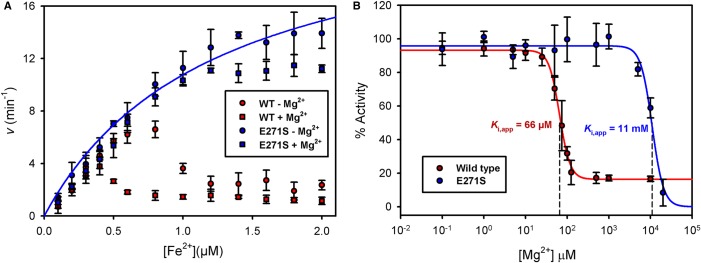
Loss of the metal-binding residue Glu271 abrogates the inhibition of *S. aureus* ferrochelatase by iron and magnesium. (**A**) *v* vs. [Fe^2+^] plots were generated for wild-type (red) and an E271S variant of *S. aureus* CpfC (blue) assayed in the presence (squares) and absence (circles) of 4 mM Mg^2+^. Fixed concentrations of 50 nM enzyme and 2 µM coproporphyrin III were used. The fitted blue line provides estimates of apparent kinetic constants when the inhibition by Fe^2+^ is removed: *k*_cat_ = 24.1 ± 2.0 min^−1^, *K*_app_(Fe^2+^) = 1.2 ± 0.2 µM. (**B**) Inhibition dose responses for wild-type (red) and an E271S variant of *S. aureus* CpfC (blue) assayed in the presence of increasing concentrations of Mg^2+^. Fixed concentrations of 50 nM enzyme, 2 µM coproporphyrin III, and 0.7 µM Fe^2+^ were used. To estimate *K*_i,app_ values, the data were fitted to a four parameter Hill equation (eqn 1, [19]) with the minimum activity (i.e. *v*_min_) constrained to a positive value.

**Figure 7.**
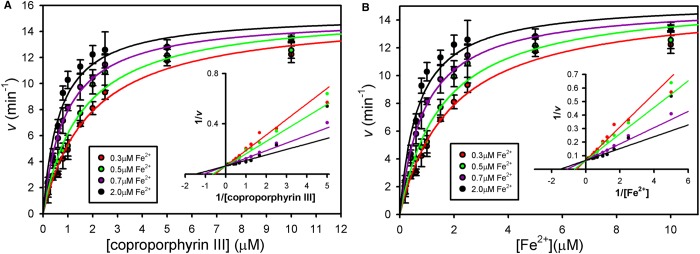
Loss of inhibitory metal site restores conventional Michaelis kinetics. Primary Michaelis–Menten plots for an E271S variant of *S. aureus* CpfC were generated with fixed concentrations of iron (**A**) and coproporphyrin III (**B**), and data were fitted to single rectangular hyperbolae via non-linear regression. Lineweaver–Burk plots (insets) are shown to highlight differences in kinetic patterns between panels (**A**) and (**B**). To avoid well-known problems with these secondary plots, trend lines were generated using the kinetic constants obtained from non-linear regression of the untransformed data.

To test the hypothesis that the metal-binding site shown in Figure 4 is involved in the Mg^2+^-mediated inhibition kinetics shown in Figure 5, Glu271 was mutated to serine and the resultant E271S mutant protein was purified and assayed in the presence and absence of Mg^2+^ (Figure 6). Figure 6A shows that Mg^2+^-potentiated inhibition was dramatically diminished in the E271S mutant, and that inhibition by Fe^2+^ in the absence of Mg^2+^ was also dramatically diminished. Figure 6B displays *K*_i,app_(Mg^2+^) values for wild-type and E271S CpfC as 66 µM and 11 mM, respectively, highlighting a dramatic loss of sensitivity to Mg^2+^ for the mutant enzyme. Together, these data strongly indicate that in wild-type *S. aureus* CpfC, the binding of Mg^2+^ or Fe^2+^ at this metal site results in enzyme inhibition. In addition, the data in Figures 4–6 suggest that loss of the inhibitory metal site should reverse the inhibitory effects of increasing iron concentrations seen in Figure 3A, so a similar kinetic study was carried out with the E271S variant of *S. aureus* CpfC including iron levels well above the inhibitory threshold for the wild-type enzyme (Figure 7). These data confirm that loss of the E271 metal site abolishes the iron-mediated inhibition with respect to coproporphyrin III (Figure 7A), and the effect of varying [coproporphyrin III] upon the kinetic constants for iron (Figure 7B) remains similar to the wild-type data (Figure 3B).

## Discussion

Preliminary kinetic analysis of *S. aureus* CpfC revealed a *k*_cat_ of 10.5 ± 0.4 min^−1^. This rate is comparable to previous measurements for other bacterial PpfCs [10], and slightly higher than previous measurements of 1.8 and 0.11 min^−1^ for CpfCs from *M. tuberculosis* and *B. subtilis*, respectively [2]. While a *k*_cat_ for *S. aureus* CpfC was recently calculated to be 165 min^−1^ via extrapolation of a substrate inhibition curve [1], the maximum rates measured (∼28 min^−1^) were of a similar magnitude to those presented herein. We report the first measurement of *K*_app_ (coproporphyrin III) for *S. aureus* CpfC as 1.5 ± 0.2 µM, which is slightly lower than previous measurements of 10.5 and 7.8 µM for CpfC enzymes from *M. tuberculosis* and *B. subtilis*, respectively [2]. Our estimate of 0.27 ± 0.04 µM for *K*_app_ (Fe^2+^) is comparable with a previous estimate of 0.6 ± 0.1 µM for this enzyme [1], although both of these measurements were hampered by substrate inhibition by iron.

Primary and secondary kinetic plots clearly demonstrate that elevating the iron concentration increases the *K*_m_ for coproporphyrin III (Figure 3A), which is consistent with Fe^2+^ causing inhibition at higher concentrations. It was therefore hypothesised that Fe^2+^ is inserted into the porphyrin substrate following binding to a well-known catalytic site adjacent to the tetrapyrrole substrate, and higher concentrations of Fe^2+^ inhibit chelation by binding at a second regulatory site. Structural modelling (Figure 4) provides convincing evidence that *S. aureus* possesses a second metal-binding site similar to the Mg^2+^-binding site in *B. subtilis* CpfC [12], although Mg^2+^ was found to potentiate Fe^2+^-mediated inhibition in the current study (Figure 5) compared with having a stimulatory role in previous reports [13]. This could be due to differences in substrates used, as the current study uses the native substrates, whereas the previous work has used Zn^2+^ and deuteroporphyrin IX as substrates [13], which could influence the behaviour of metals in the active site. Indeed, previous measurements of *S. aureus* CpfC kinetics demonstrate a lack of Fe^2+^-mediated inhibition when protoporphyrin IX is used as a substrate [1].

**Figure 8.**
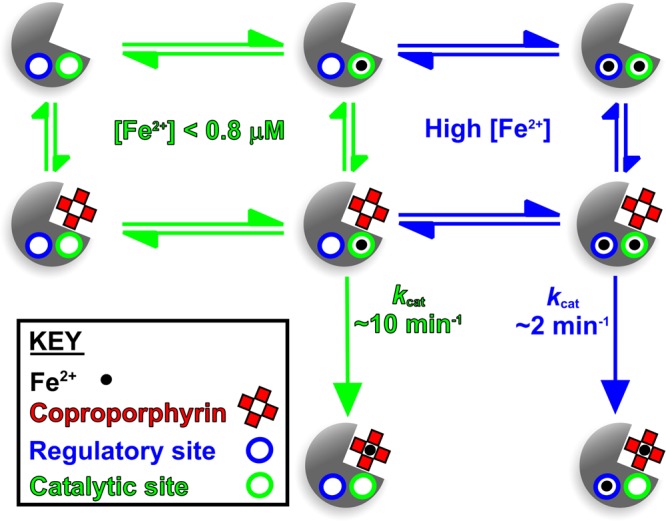
Kinetic model for iron-dependent inhibition of *S. aureus* ferrochelatase. Processes shown in blue and green depict conditions of high- and low-iron concentrations, where the inhibitory regulatory site is occupied or empty, respectively. Since inhibition is only observed at higher iron concentrations, it is unlikely that the regulatory site would be occupied when the catalytic site is empty, so these intermediates are not shown.

To confirm the involvement of the regulatory metal site (Figure 4) in allosteric modulation of *S. aureus* CpfC, the E271S mutant enzyme was purified and kinetic analyses were performed in the presence and absence of Mg^2+^. A high concentration of 4 mM Mg^2+^ was used initially to saturate metal binding (i.e. Fe^2+^ and Mg^2+^) at the inhibitory site. Substitution of this glutamate residue with serine resulted in two dramatic outcomes: substrate inhibition by Fe^2+^ was lost and Mg^2+^ was no longer a potent inhibitor of metal chelation (Figure 6A). This strongly suggests that the binding of Fe^2+^ or Mg^2+^ is stabilised by Glu271. To add weight to this conclusion, Mg^2+^ dose–response data confirm that Mg^2+^ is a potent inhibitor of the wild-type enzyme but not the E271S mutant (Figure 6B). Steady-state kinetics for the E271S mutant revealed that loss of metal binding at this residue reverses the Fe^2+^-mediated increase in *K*_m_(coproporphyrin III) (Figure 7A) that is seen for the wild-type enzyme in Figure 3A. The effect of varying [coproporphyrin III] upon the kinetic constants for iron elicits the same pattern for the wild-type (Figure 3B) and E271S mutant (Figure 7B), which strongly suggests that metal binding at the E271S site largely inhibits coproprophyrin binding and/or chelation rather than iron binding at the catalytic site. Taken together, all data presented herein support the model for wild-type *S. aureus* CpfC in Figure 8 where iron binding to a lower affinity regulatory site at E271 (blue) diminishes the rate of iron chelation at an inner catalytic site (green) via increasing the *K*_m_ for the coproporphyrin III substrate.

It is well-known that there is fierce competition for iron both during infection resulting from nutritional immunity [22], and during growth in mixed microbial communities mediated by microbial siderophores. Therefore, where iron is scarce, it is important for *S. aureus* to tightly regulate how this nutrient is used within the cell. The current work is consistent with a model whereby at very low Fe^2+^ concentrations ferrochelatase is able to insert iron into coproporphyrin III, whereas at higher iron concentrations flux through this step of the pathway is down-regulated. There may be many reasons for this substrate inhibition, including avoiding accumulation of Fe-coproporphyrin III, or perhaps once the haem requirements of the cell are satisfied Fe^2+^ is made available for other processes (e.g. Fe–S cluster assembly). Future studies on bacteria that perform coproporphyrin-dependent haem biosynthesis are required to reveal the hierarchical channelling of iron for different uses in the cell, which will be important during infection (*S. aureus*, *M. tuberculosis*, and *P. acnes*) and growth in microbial communities (*B. subtilis*).

## Abbreviations

CgoX: coproporphyrinogen oxidase
ChdC: coprohaem decarboxylase
CpfC: coproporphyrin ferrochelatase
PpfC: protoporphyrin ferrochelatase
